# Case report: Superficial soft tissue implantation metastases of oncocytic thyroid carcinoma: presentation of two complicated cases

**DOI:** 10.3389/fmed.2026.1933710

**Published:** 2026-10-29

**Authors:** Lei Tang, Xueyong Zheng, Qihang Li, Jing Shen

**Affiliations:** 1 Department of Thyroid Surgery, The Second Affiliated Hospital of Jiaxing University, Jiaxing, China; 2 Department of Pathology, The Second Affiliated Hospital of Jiaxing University, Jiaxing, China; 3 Department of Breast Surgery, The Second Affiliated Hospital of Jiaxing University, Jiaxing, China

**Keywords:** case report, diagnostic, implantation metastasis, oncocytic carcinoma, thyroid cancer

## Abstract

**Background:**

Superficial soft tissue implantation metastases of oncocytic thyroid carcinoma are rare, especially following open surgeries, and are frequently misdiagnosed or overlooked. The objective of this study was to explore more characteristics of such cases, aiming to improve diagnostic accuracy.

**Case presentations:**

Herein, we describe two middle-aged women with a history of prior thyroid surgery. One patient underwent repeated thyroid procedures; an initial diagnosis of follicular tumor of uncertain malignant potential (FT-UMP) was revised to oncocytic thyroid carcinoma on final histopathological examination. The second patient had a single prior thyroid operation, and oncocytic thyroid carcinoma was identified during evaluation for bone metastases. Contrast-enhanced CT revealed multiple cervical soft tissue implantation metastases in both patients.

**Conclusion:**

These cases highlight the diagnostic pitfalls of implantation metastases from oncocytic thyroid carcinoma and underscore the importance of thorough pathological and surgical evaluation for patients with suspected recurrent disease.

## Introduction

Implantation metastases of thyroid carcinoma are rare but may occur following fine needle aspiration cytology (FNAC) or endoscopic thyroid surgery (1–6); this complication is seldom reported after open thyroid procedures. Misdiagnosis of such cases can undoubtedly lead to tumor recurrence and repeated surgery, further raising the risk of surgical-related complications.

The first patient initially presented with a painless anterior neck mass. In contrast, the second patient was evaluated after resection of a pelvic lesion histopathologically confirmed as bone metastasis secondary to thyroid carcinoma. Multiple soft tissue implantation metastases in the cervical region were identified on contrast-enhanced CT in both patients. Neck masses encompass a broad spectrum of etiologies, including infectious, congenital, and neoplastic lesions (7). For patients with a history of prior thyroid surgery — one who underwent repeated thyroid operations and the other with a single previous thyroid procedure — the differential diagnosis must also include tumor seeding, a rare but clinically significant possibility. These cases underscore the critical need to consider implantation metastases in such scenarios, particularly when evaluating recurrent lesions in patients with a history of thyroid surgery. Herein, we report these two complicated cases of oncocytic thyroid carcinoma with superficial soft tissue implantation metastases.

This study provides a comprehensive analysis of diagnostic and therapeutic management of superficial soft tissue implantation metastases arising from oncocytic thyroid carcinoma. By exploring this rare clinical entity, we aim to enhance recognition among clinicians and pathologists, thereby reducing diagnostic delays and optimizing treatment strategies. The study protocol received approval from the Institutional Ethics Committee of the Second Affiliated Hospital of Jiaxing University.

## Case presentation

### Case 1

A 57-year-old woman was referred to our thyroid surgery outpatient clinic with a chief complaint of a gradually enlarging, painless anterior neck mass that had been present for approximately two years. The patient first underwent thyroid surgery at age 19, followed by a second thyroid procedure over a decade prior to the current presentation. According to the patient's report, histological examination following both prior surgeries had indicated benign pathology.

The mass demonstrated progressive growth but remained asymptomatic, with no associated dysphagia, hoarseness, or compressive symptoms. Physical examination revealed a left-sided visible swelling (approximately 1.0 × 0.5 cm). Palpation identified a firm, non-tender mass with regular borders anterior to the trachea. No bruit was auscultated over the mass. The puncture result showed an epithelial-derived neoplastic lesion (oncocytoma?). The thyroglobulin level was 48.50 ng/mL. We performed a third operation, during which the lump was completely excised. Intraoperative pathology indicated a FT-UMP, as no definitive capsular or vascular invasion was identified within the limited sampled sections, prompting us to conclude the procedure. Final histopathology, however, presented oncocytic thyroid carcinoma, widely invasive type, with vascular invasion (+) (Figure 1). Immunohistochemistry: Galectin (rarely positive), CD31-positive vascular invasion, ERG (negative), TTF-1 (focal positive), PAX-8 (positive), GATA3 (negative), PTH (negative) (Figure 2).

**Figure 1:**
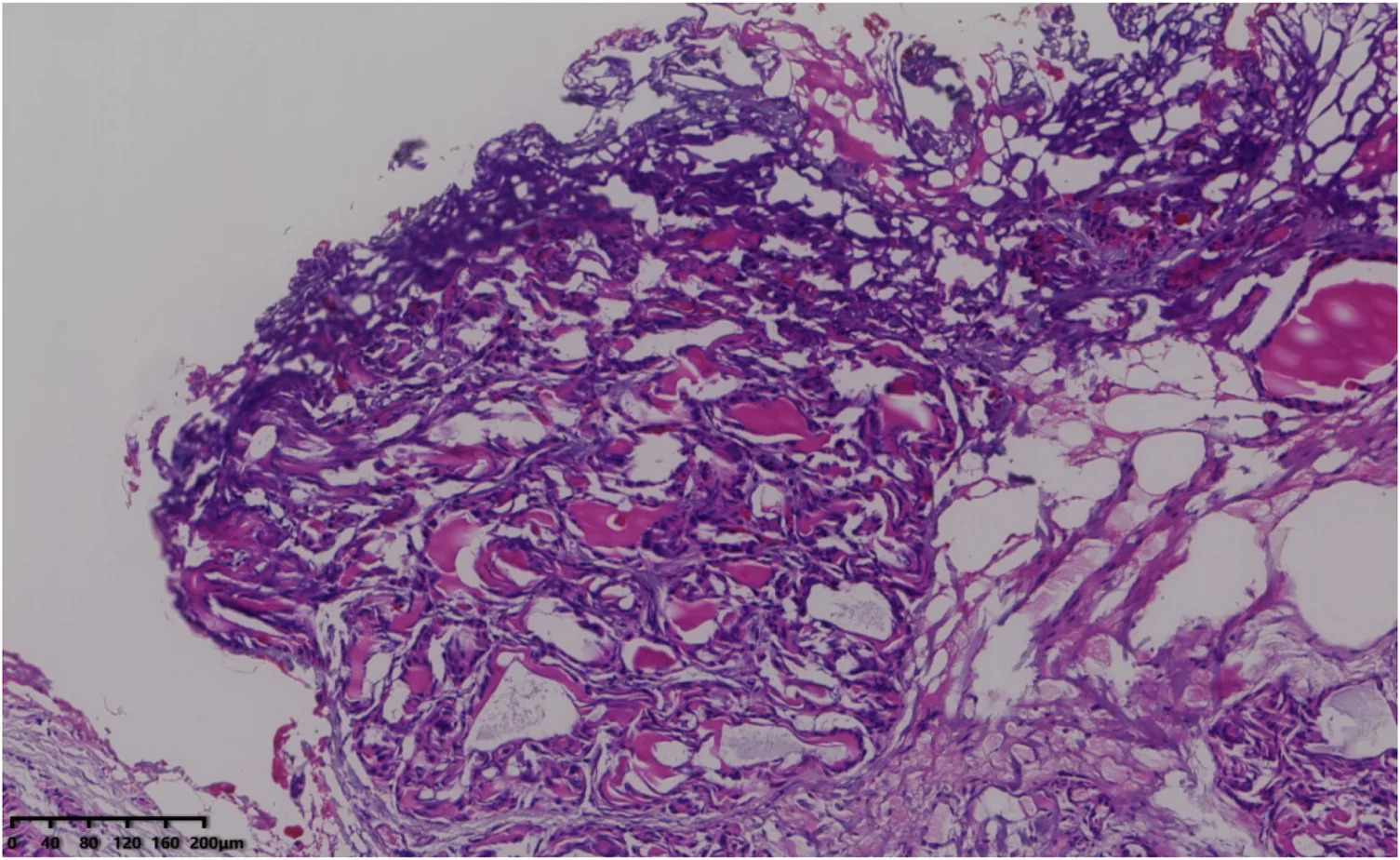
Histopathology of the mass following operation demonstrating oncocytic thyroid carcinoma. (HE*10). HE, Hematoxylin-Eosin staining.

**Figure 2:**
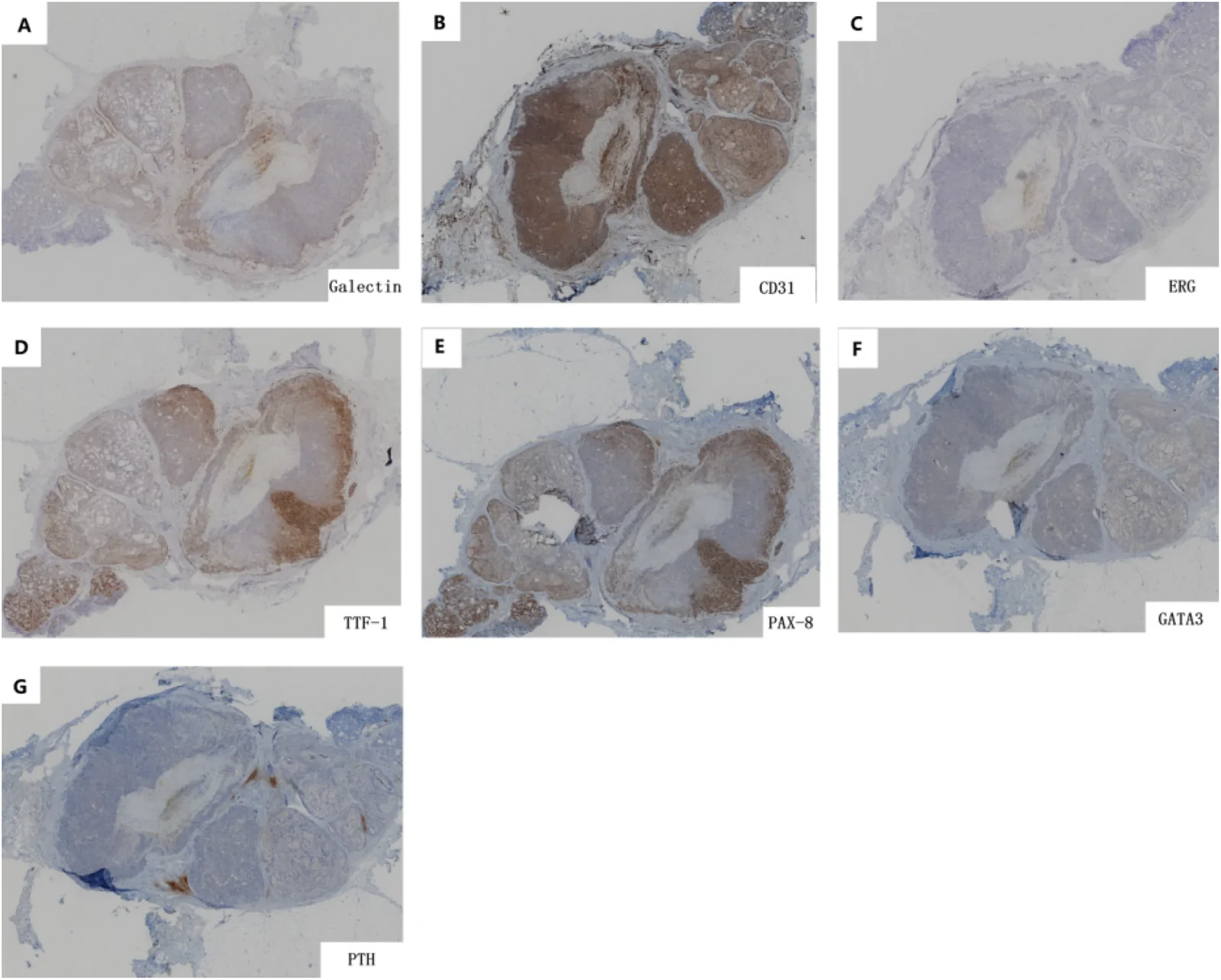
Immunohistochemistry of the tumor. **(A)** Galectin, **(B)** CD31, **(C)** ERG, **(D)** TTF-1, **(E)** PAX-8, **(F)** GATA3, **(G)** PTH.

Therefore, we hypothesized that this anterior-neck mass might represent soft tissue implantation metastases following the initial thyroid surgery, which raised our concern about the pathological evaluation of the primary tumor. Consequently, original pathological slides from the prior surgery were obtained for further consultation. The pathological consultation results revealed thyroid follicular proliferation with focal suspicious capsular invasion, consistent with a FT-UMP. Owing to insufficient residual paraffin-embedded tissue of the primary tumor for molecular testing, clonal relatedness between the two lesions was inferred based on morphological and immunohistochemical similarity.

Our subsequent diagnostic evaluation incorporated contrast-enhanced neck CT, 116-gene panel analysis for thyroid carcinoma, positron emission tomography-computed tomography (PET-CT), and electronic nasopharyngolaryngoscopy. Comparative analysis between the current contrast-enhanced neck CT and prior non-contrast imaging revealed multiple new lesions consistent with implantation metastases (Figure 3). The 116-gene panel analysis revealed the following somatic variants: TERT promoter mutation (c.-124C＞T); NRAS missense mutation (c.182A＞G p.Q61R exon3); ARID1B missense mutation (c.6338G＞T p.R2113L exon 20); RBM10 frameshift mutation (c.1462_1471del p.A488Cfs*213 exon14).

**Figure 3:**
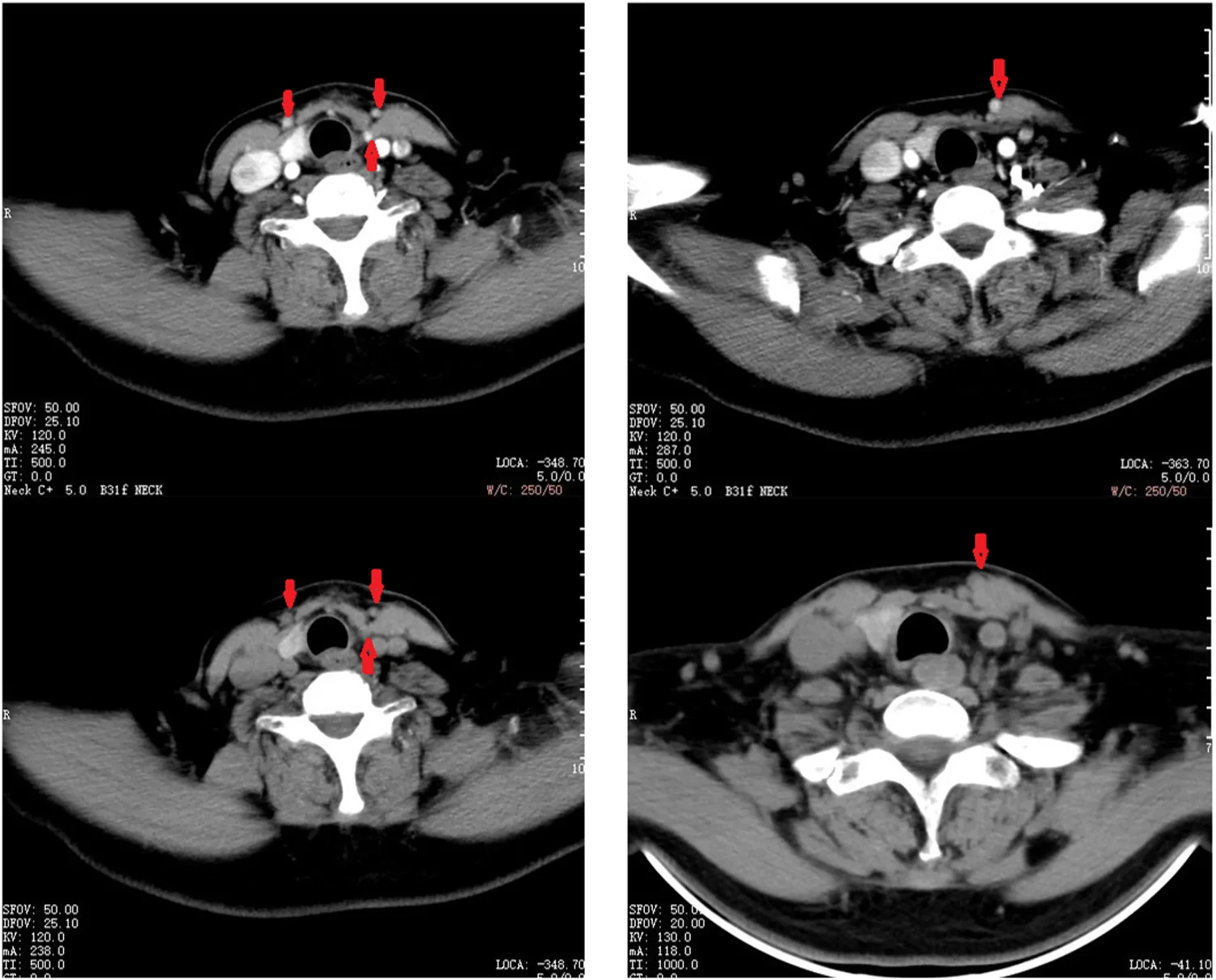
Contrast-enhanced versus non-contrast neck images of case 1.

PET-CT demonstrated no evidence of hypermetabolic activity suggestive of distant metastasis. Electronic nasopharyngolaryngoscopy identified a nasopharyngeal bulge and right pyriform sinus mass; however, the patient declined biopsy of these lesions.

### Treatment and prognosis

Postoperative enhanced CT demonstrated multiple superficial soft tissue implantation metastases in the medial aspects of both sternocleidomastoid muscles and the anterior strap muscle region, following which completion thyroidectomy and left lateral cervical lymph node dissection (level Ⅱ, Ⅲ, Ⅳ) were carried out (Figure 4). Final Pathological Diagnosis: (1)Metastatic carcinoma in 1/3 lymph node (metastatic lesions larger than 2 mm and smaller than 3 cm), Level VI (left); (2) 4 tumor nodules were identified in anterior cervical soft tissue [consistent with CT findings (Figures 3)]. Comparison of contrast-enhanced and non-contrast neck images demonstrates improved hilar definition in tumor nodules. We recommend subsequent radioactive iodine (RAI) therapy as adjuvant treatment.

**Figure 4:**
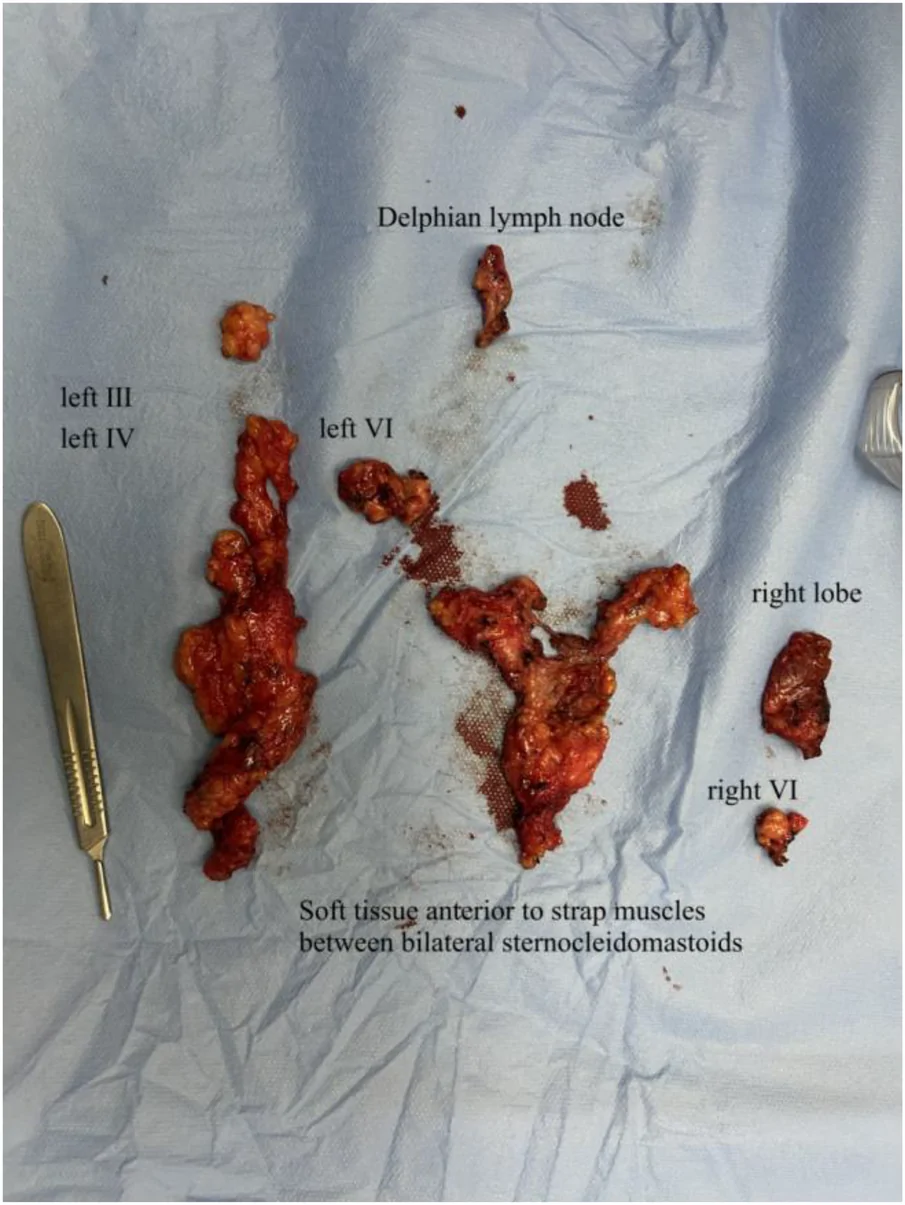
The surgical resection specimen.

The preoperative laboratory findings of this case were all within normal ranges, including: thyroglobulin (TG), thyroid function (with daily levothyroxine 50 μg supplementation post-second surgery), anti-thyroglobulin antibody (TgAb), anti-thyroid peroxidase antibody (TPOAb), parathyroid hormone (PTH). Notably, no significant postoperative decline in PTH levels was observed, indicating preserved parathyroid function.

The patient was discharged after surgery without complications and was followed up for 4 months via outpatient visits. Serial thyroglobulin measurements showed a progressive decline from 48.50 ng/mL preoperatively to 38.51 ng/mL postoperatively, and further to 6.48 ng/mL at the most recent follow-up one month ago. Long-term surveillance with periodic clinical exams and thyroglobulin monitoring is planned. At the last follow-up, the patient remained recurrence-free with normal thyroid function (thyroid-stimulating hormone (TSH): 3.602 *μ*IU/L, free T3: 4.16 pmol/L, free T4: 12.27 pmol/L).

### Case 2

A 59-year-old woman had undergone partial right thyroidectomy more than 10 years previously, and postoperative histopathology had confirmed benign thyroid disease. She presented with a 1-year history of persistent left hip discomfort that had progressively worsened over the preceding two months. Preoperative pelvic magnetic resonance imaging (MRI) demonstrated focal osteolytic destruction of the left ilium associated with a soft tissue mass suspicious for malignancy, as well as a suspicious lesion in the proximal right femur;these imaging findings favored metastatic bone malignancy rather than a primary bone tumor.

Subsequent PET-CT detected a heterogeneously hypodense FDG-avid nodule at the inferior pole of the left thyroid lobe and mild FDG uptake in a single level VI cervical lymph node. Multifocal FDG-hypermetabolic osseous lesions involved the C7 vertebral body, left T1 vertebral appendage, proximal right femur, and left ilium, with osteolysis and soft tissue mass formation at the left iliac site. The composite imaging findings were highly suggestive of thyroid carcinoma with disseminated bone metastases.

On July 30, 2025, the patient underwent resection of the pelvic bone lesion. Preoperative suspicion of metastatic thyroid carcinoma was ultimately confirmed by immunohistochemical evaluation of the pelvic surgical specimen in the context of her complete clinical history. All resection margins of the pelvic lesion, including bilateral, superior, inferior, and cutaneous margins, were negative for tumor infiltration. Immunohistochemical profiling of the pelvic lesion yielded the following results: Galectin-3 (negative), CK19 (positive), BRAF V600E (partial positive), CD56 (focal negative), HBME1 (positive), PAX-8 (positive), TTF-1 (positive), NKX3.1 (negative).

Total thyroidectomy with bilateral level VI lymph node dissection was performed on January 14, 2026. Preoperative TG: 206.70 ng/mL. No substantial reduction in TG was observed in the immediate postoperative period; however, a downward TG trend emerged two months after surgery, falling to 3.68 ng/mL at the four-month postoperative follow-up. The preoperative parathyroid hormone (PTH) concentration was 34.98 pg/mL. PTH decreased to 9.12 pg/mL on postoperative day 1 and returned to the normal range (20.83 pg/mL) at the one-month follow-up.Pathological examination revealed diffuse invasive oncocytic thyroid carcinoma measuring 1.8 × 1.8 × 1.5 cm within the left thyroid lobe (Figure 5), whereas the right thyroid lobe showed nodular goiter. The subcutaneous cervical nodule visualized on CT (Figure 6) was morphologically consistent with infiltrative or metastatic thyroid carcinoma when correlated with clinical history. All 20 resected right level VI lymph nodes were negative for malignancy, whereas metastatic carcinoma was detected in 1 of 3 harvested left level VI lymph nodes. The final pathological TNM staging was pT4N1aM1. Immunohistochemical profiling yielded the following results for the primary thyroid tumor: Galectin-3 (negative), CK19 (positive), BRAF V600E (positive), Cyclin D1 (positive), CD56 (positive), HBME1 (positive), with CD34 highlighting vascular endothelium. Apart from focal CD56 loss in the hip bone metastasis, the rest of the immunohistochemical markers were largely consistent between the primary tumor and the prior left hip lesion, confirming their common clonal origin.

**Figure 5:**
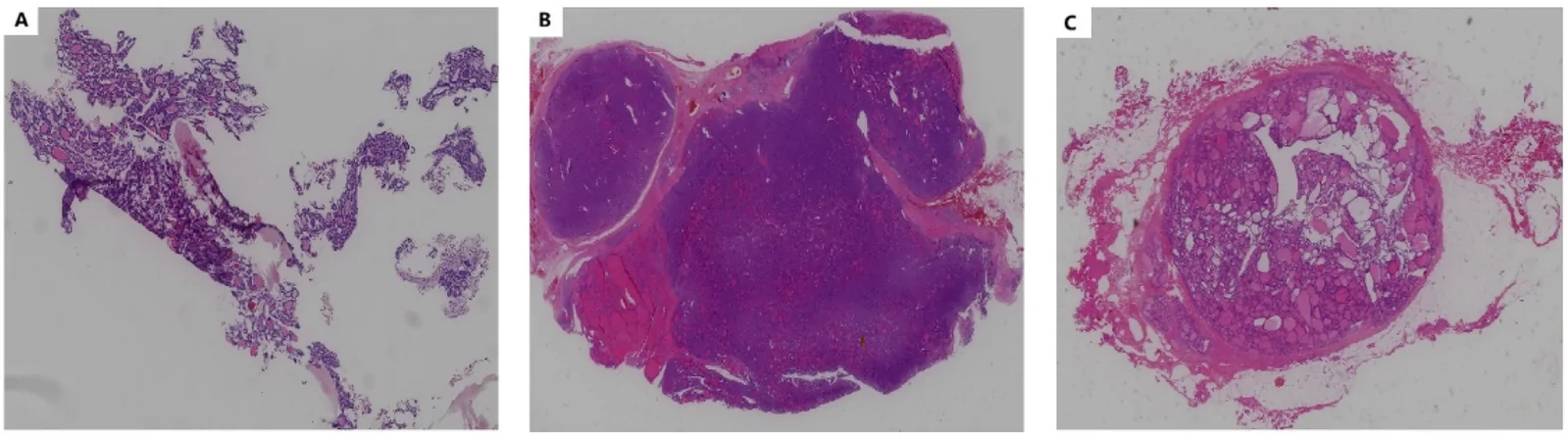
Histopathological comparison of the three lesions.**(A)** Initial iliac-bone metastatic focus. **(B)** Primary oncocytic thyroid carcinoma. **(C)** Cervical subcutaneous implantation-metastatic nodule.

**Figure 6:**
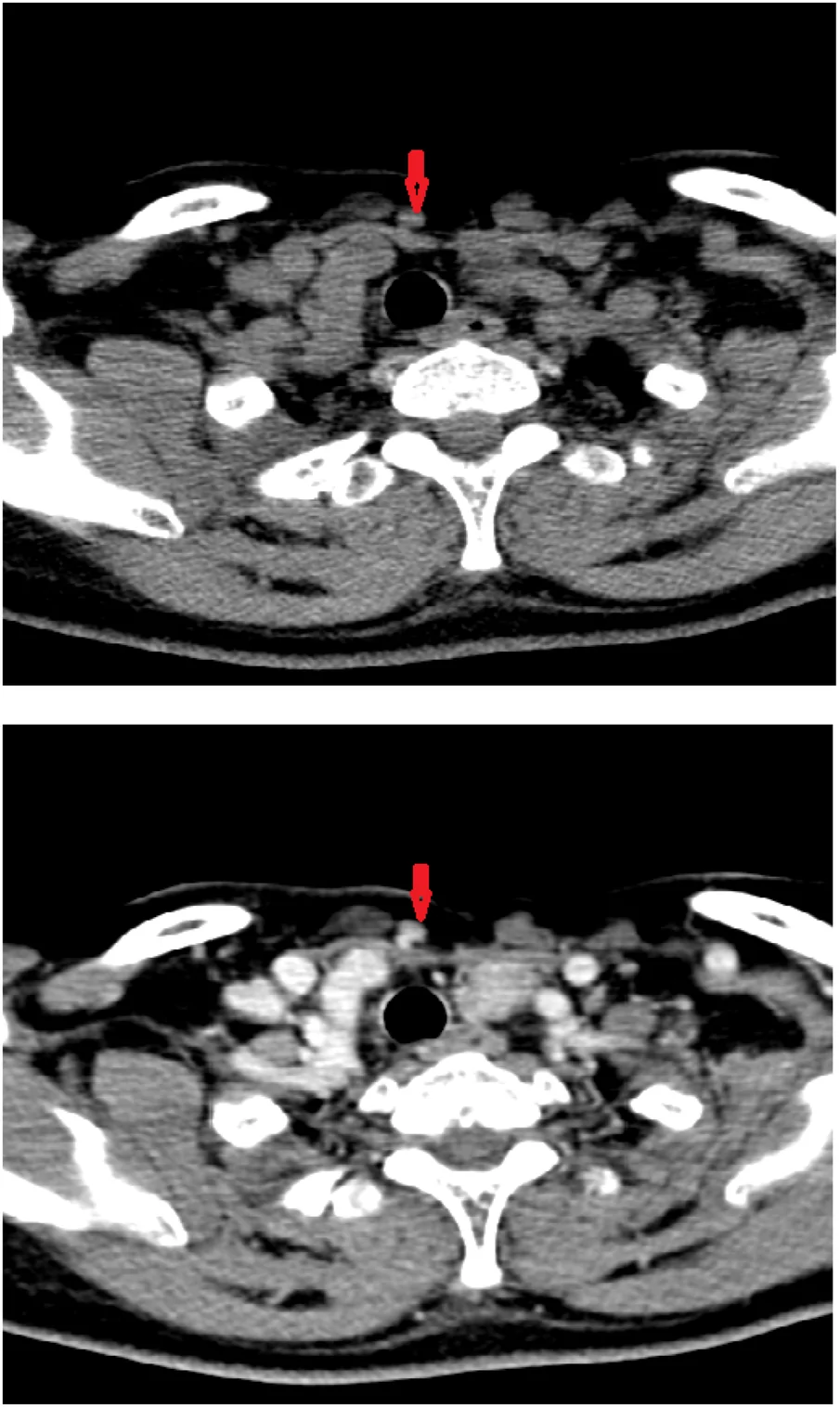
Contrast-enhanced versus non-contrast neck images of case 2.

## Discussion

Oncocytic thyroid carcinoma represents a relatively uncommon histological subtype of thyroid malignancy, accounting for approximately 5% of all thyroid cancers and exhibiting a comparatively low incidence of lymph node metastasis. In the present cases, the secondary lesions were located adjacent to the surgical incision, strictly confined within the previous operative field and outside the residual thyroid parenchyma, with no histological evidence of continuous infiltrative growth extending from the thyroid remnant. These findings were highly consistent with the surgical approach and operative field of the prior procedure. These lesions presented as solitary nodules without evident surrounding vascular invasion. Histologically, no identifiable lymphatic channels were observed within the perilesional stroma of the secondary lesions. Meanwhile, no distant visceral metastases were detected on preoperative cross-sectional imaging, rendering lymphatic and hematogenous dissemination less likely. Despite an interval of more than ten years between the two surgeries in the first patient, the aforementioned characteristics strongly suggested that these metastases represented implantation-related secondary lesions originating from prior surgical manipulation.

Table 1 summarizes previously reported cases of cervical subcutaneous metastases, including etiologies, locations, intervals from primary surgery to metastasis, and clinical outcomes, providing a contextual framework for comparison with the present cases. As cervical subcutaneous metastases remain a rare clinical entity, distinguishing these lesionsfrom hematogenous spread is critical for guiding clinical decision-making—previous reports predominantly link such metastases to procedural implantation, as reflected in Table 1. In contrast, hematogenous spread typically presents with distant organ involvement (e.g., liver, lung) rather than isolated subcutaneous nodules, which aligns with the current case's focus on cervical subcutaneous lesions without evidence of systemic hematogenous dissemination. The long interval from primary treatment to metastasis in previous cases further supports the indolent nature of implantation metastases (8–10). Similar to those historical reports, our two patients developed subcutaneous cervical lesions more than ten years after initial thyroid surgery; no concurrent distant visceral metastases were identified, which further favors a diagnosis of surgical implantation-associated metastasis.

**Table 1:** Previously reported cervical subcutaneous metastasis cases.

Author (Year)	Is implantation metastasis?	Location	Interval	Outcome
Harach et al., 2004 (8)	Yes	Left side of the neck	14 years	Unclear
Bongiovanni et al., 2016 (9)	Yes	Cervical subcutaneous nodule (0.5/1.0 cm))	60 months	Liver and sternum metastases
Bongiovanni et al., 2016 (9)	Yes	Cervical subcutaneous nodule (0.9 cm)	59 months	Died after liver, brain and cardiac metastases
Bongiovanni et al., 2016 (9)	Yes	Cervical subcutaneous nodule near surgical incision	36 months	Lung metastasis
Chiapponi et al., 2022 (10)	Unclear	7 (13.7%) of 51 patients with isolated cervical tumors	36-211moths	2 patients (28.6%) achieved excellent response at last follow-up. 5 (71.4%) had indeterminate (*n* = 2), biochemical incomplete (*n* = 2), or structural incomplete response (*n* = 1, stable lung metastases at 68-month follow-up).

Given the diagnostic confirmation of implantation metastases, surgical management for such rare lesions warrants careful risk-benefit evaluation. Total thyroidectomy constitutes the most frequent surgical procedure for oncocytic thyroid carcinoma, whereas neck dissection is generally performed only in the setting of clinically apparent positive cervical lymph nodes. Given the intraoperative diagnosis of FT-UMPand patient 1's history of multiple thyroid operations, extended further surgery would offer limited benefit while substantially increasing risks including recurrent laryngeal nerve injury and parathyroid dysfunction, raising concerns regarding overtreatment. Postoperative final histopathology confirmed widely-invasive oncocytic thyroid carcinoma with vascular invasion (+). Subsequent cervical contrast-enhanced CT demonstrated multiple implantation metastases; therefore, a fourth surgical intervention was performed.

Histologically, the tumor showed classic oncocytic features. Immunohistochemically, strong PAX-8 positivity together with focal TTF-1 positivity confirmed thyroid origin. Negative staining for GATA3, PTH and ERG effectively excluded differential diagnoses. CD31 immunostaining highlighted vascular invasion, an adverse prognostic feature. Collectively, these findings confirmed the diagnosis of oncocytic thyroid carcinoma. Molecular testing identified concurrent RBM10 and ARID1B mutations. Notably, ARID-family gene mutations, which encode components of SWI/SNF chromatin-remodelling complexes, are detected in approximately 9% of papillary thyroid carcinomas, including ARID1A (3%), ARID2 (2%), and ARID1B (1%) (11). Likewise, loss-of-function alterations or mutations in RBM10 promote thyroid cancer metastasis via aberrant regulation of RNA splicing (12). This co-occurrence of RBM10 and ARID1B mutations has, to our knowledge, not been previously reported in oncocytic thyroid carcinoma, suggesting a potential cooperative association with driving tumor progression and aggressive behavior.

RAI therapy is reported 54% of patients, yet there remains no general agreement regarding its clinical benefits (13). For recurrent or metastatic oncocytic thyroid carcinoma, radioactive iodine (RAI) is recognized as a treatment option in the latest American Thyroid Association (ATA) and NCCN Thyroid Cancer Guidelines (14). Several studies report that oncocytic carcinomas exhibit lower iodine avidity and worse prognosis (13, 15, 16). Additionally, TERT mutations have been investigated as a risk factor for the recurrence of central and lateral lymph nodes (17–19). Meanwhile, TERT gene mutation can guide the treatment of oncocytic carcinoma at the molecular level (20). Complementary siTERT gene therapy demonstrates synergistic enhancement of antitumor effects when combined with TERT-targeted I-131 therapy, representing a promising novel treatment paradigm (21). Supporting the importance of genetic profiling, Lu et al. reported a oncocytic thyroid carcinoma case harboring an NRAS Q61 K mutation that exhibited favorable radioiodine (RAI) avidity and treatment response (22). Given this patient's aggressive disease course (multiple recurrences) and high-risk molecular profile (TERT promoter + NRAS mutations), we recommend RAI therapy, notwithstanding the limited evidence for I-131 efficacy in oncocytic carcinoma. Nevertheless, RAI therapy was not ultimately administered owing to patient refusal.

The identification of two cases of cervical soft tissue implantation metastases in our center carries important clinical implications for follow-up practice. We reflected on whether the comprehensive preoperative work-up performed for the fourth surgery of Case 1 should have been implemented prior to the third operation, to detect soft tissue implantation metastases at an earlier stage. While surgical implantation is an uncommon metastatic route for oncocytic thyroid carcinoma, the coexistence of two such cases in our center suggests this manifestation may not be as rare as believed. However, broad examinations including a 116-gene panel, PET-CT and contrast-enhanced neck CT for a single neck mass may lead to overmedicalization, which necessitates rigorous risk-benefit evaluation. Superficial implantation lesions in both cases are easily missed, so clinicians must remain highly vigilant during follow-up for patients with resected oncocytic thyroid carcinoma.

## Data Availability

The original contributions presented in the study are included in the article/Supplementary Material, further inquiries can be directed to the corresponding author.
